# Supplementary material for: Spectroscopic and Structural Study of Some Oligosilanylalkyne Complexes of Cobalt, Molybdenum and Nickel
Source: Molecules. 2019 Jan 8;24(1):205. doi: 10.3390/molecules24010205 (PMC6337173; doi:10.3390/molecules24010205)
Supplement: Supplementary file 1 [file molecules-24-00205-s001.pdf]

Table S1. Crystallographic data for compounds **2b**, **7a**, **11a**, **13c**, and **dippeNiCOD**

	**2b**	**7a**	**11a**	**13c**	**dippeNiCOD**
Empirical formula	$C_{25}H_{38}Mo_2O_4Si_4$	$C_{23}H_{32}Co_2GeO_6Si_3$	$C_{42}H_{60}Co_4Si_8O_{12}S_2$	$C_{23}H_{44}NiSP_2Si$	$C_{22}H_{44}NiP_2$
M_w	706.79	679.21	1281.46	501.38	429.22
Temperature [K]	100(2)	100(2)	100(2)	100(2)	100(2)
Size [mm]	0.32×0.28×0.15	0.42×0.36×0.26	0.34×0.22×0.22	0.34×0.28×0.20	0.35×0.27×0.22
Crystal system	triclinic	monoclinic	monoclinic	monoclinic	monoclinic
Space group	P-1	P2(1)	P2(1)/n	P2(1)/c	C2/c
a [Å]	9.605(3)	9.634(2)	19.710(4)	10.586(2)	17.327(3)
b [Å]	10.237(2)	9.886(2)	16.305(3)	13.380(3)	9.640(2)
c [Å]	17.335(6)	15.986(3)	20.301(4)	19.599(4)	13.539(3)
α [°]	85.09(3)	90	90	90	90
β [°]	75.13(3)	91.91(3)	113.05(3)	103.28(3)	96.63(3)
γ [°]	71.15(3)	90	90	90	90
V [$Å^3$]	1559(2)	1522(2)	6003(2)	2702(2)	2246(3)
Z	2	2	4	4	4
ρ_{calc} [gcm^{-3}]	1.506	1.482	1.418	1.233	1.269
Absorption coefficient [mm^{-1}]	0.985	2.209	1.366	0.966	1.010
F(000)	720	692	2640	1080	936
θ range	$2.10<\theta<26.37$	$2.12<\theta<26.36$	$1.66<\theta<26.37$	$1.86<\theta<26.30$	$2.37<\theta<26.34$
Reflections collected/unique	11088/6056	12117/6042	47085/12247	21121/5466	8694/2282
Completeness to θ [%]	95.0	99.6	99.7	99.7	99.7
Data/restraints/parameters	6056/0/325	6042/1/326	12247/12/650	5466/0/264	2282/0/118
Goodness of fit on F^2	1.03	0.97	1.11	1.11	1.14
Final R indices [$I>2\sigma(I)$]	R1=0.075, *w*R2=0.178	R1=0.023, *w*R2=0.050	R1=0.069, *w*R2=0.111	R1=0.078, *w*R2=0.148	R1=0.038, *w*R2=0.089
R indices (all data)	R1=0.088, *w*R2=0.184	R1=0.024, *w*R2=0.050	R1=0.106, *w*R2=0.121	R1=0.113, *w*R2=0.162	R1=0.040, *w*R2=0.090
Largest diff. Peak/hole [e^-/ $Å^3$]	1.65/-1.19	0.58/-0.30	0.63/-0.48	0.66/-0.74	0.64/-0.26
